# Supplementary material for: Phosphodiester modifications in mRNA poly(A) tail prevent deadenylation without compromising protein expression
Source: RNA. 2020 Dec;26(12):1815–37. doi: 10.1261/rna.077099.120 (PMC7668260; doi:10.1261/rna.077099.120)
Supplement: Supplemental Material [file supp_26_12_1815__index.html]

Phosphodiester modifications in mRNA poly(A) tail prevent deadenylation without compromising protein expression — Supplemental Material 

# Phosphodiester modifications in mRNA poly(A) tail prevent deadenylation without compromising protein expression

## Supplemental Material

- Supplemental\_Material.docx
