# Supplementary material for: Phosphodiester modifications in mRNA poly(A) tail prevent deadenylation without compromising protein expression
Source: RNA. 2020 Dec;26(12):1815–37. doi: 10.1261/rna.077099.120 (PMC7668260; doi:10.1261/rna.077099.120)
Supplement: Supplemental Material [file supp_077099.120_Supplemental_Material.docx]

SUPPLEMENTARY DATA

**Phosphodiester modifications in mRNA polyA tail prevent deadenylation without compromising protein expression**

Dominika Strzelecka,^1,#^ Miroslaw Smietanski,^2,#^ Pawel J. Sikorski,^2^ Marcin Warminski,^1^ Joanna Kowalska,^1,^* Jacek Jemielity^2,*^

^1^ Division of Biophysics, Institute of Experimental Physics, Faculty of Physics, University of Warsaw, Pasteura 5, 02-093 Warsaw, Poland

^2^ Centre of New Technologies, University of Warsaw, Banacha 2C, 02-097 Warsaw, Poland

# These authors contributed equally to the work

* To whom correspondence should be addressed. Tel: +48 22 55 43774; Fax: +48 22 55 43771; Email: jkowalska@fuw.edu.pl, j.jemielity@cent.uw.edu.pl

List of content

[Supplementary Tables 2](#_Toc48143986)

[Supplementary Figures 24](#_Toc48143987)

[Experimental procedures 45](#_Toc48143988)

[Synthesis of reference compounds and internal standards 45](#_Toc48143989)

[Solid-phase synthesis of oligoribonucleotide 5’-phosphate pG(CU)_5_A_11_ used as a control sample (RNA C, Table 1) 46](#_Toc48143990)

[Purification of human CNOT7 deadenylase 47](#_Toc48143991)

[References 47](#_Toc48143992)

# Supplementary Tables

*Table S1. MS parameters and selected MRM pairs for each analytes.*

| Analyte | MS Transition (m/z)  Q1 Q2 | | Declastering Potential (V) | Collision Energy (V) | Retention Time (min) |
| --- | --- | --- | --- | --- | --- |
| AMP_1 | 346.0 | 78.8 | -55 | -34 | 9.0 |
| AMP_2 | 346.0 | 96.8 | -55 | -62 | 9.0 |
| AMP_3 | 346.0 | 134.2 | -55 | -38 | 9.0 |
| [^18^O. ^18^O] AMP_1 | 350.0 | 82.8 | -55 | -34 | 9.0 |
| [^18^O. ^18^O] AMP_2 | 350.0 | 100.9 | -55 | -62 | 9.0 |
| [^18^O. ^18^O] AMp_3 | 350.0 | 134.0 | -55 | -42 | 9.0 |
| ^D^AMP_1 | 347.0 | 78.8 | -55 | -34 | 9.0 |
| ^D^AMP_2 | 347.0 | 96.8 | -55 | -62 | 9.0 |
| ^D^AMP_3 | 347.0 | 135.2 | -55 | -38 | 9.0 |
| AMPS_1 | 362.0 | 94.7 | -55 | -34 | 10.1 |
| AMPS_2 | 362.0 | 79.0 | -55 | -62 | 10.1 |
| AMPS_3 | 362.0 | 133.9 | -55 | -38 | 10.1 |
| [^18^O. ^18^O] AMPS_1 | 366.0 | 98.9 | -55 | -34 | 10.1 |
| [^18^O. ^18^O] AMPS_2 | 366.0 | 83.0 | -55 | -62 | 10.1 |
| [^18^O. ^18^O] AMPS_3 | 366.0 | 133.9 | -55 | -42 | 10.1 |
| GMP_1 | 362.0 | 79.0 | -55 | -62 | 8.4 |
| GMP_2 | 362.0 | 150.0 | -55 | -38 | 8.4 |
| [^18^O. ^18^O] GMP_1 | 366.0 | 83.0 | -55 | -62 | 8.4 |
| [^18^O. ^18^O] GMP_2 | 366.0 | 80.9 | -55 | -60 | 8.4 |
| [^18^O. ^18^O] GMP_3 | 366.0 | 101.0 | -55 | -60 | 8.4 |
| AMPH_1 | 329.9 | 134.2 | -45 | -38 | 8.7 |
| AMPH_2 | 329.9 | 63.0 | -45 | -38 | 8.7 |
| AMPH_3 | 329.9 | 80.9 | -45 | -52 | 8.7 |
| [^18^O. ^18^O] AMPH_1 | 334.0 | 67.0 | -40 | -36 | 8.7 |
| [^18^O. ^18^O] AMPH_2 | 334.0 | 134.1 | -45 | -38 | 8.7 |
| [^18^O. ^18^O] AMPH_3 | 334.0 | 84.8 | -40 | -52 | 8.7 |
| AMPBH_3__1 | 344.0 | 63.0 | -45 | -66 | 9.5 |
| AMPBH_3__2 | 344.0 | 134.1 | -45 | -38 | 9.5 |
| AMPBH_3__3 | 344.0 | 76.9 | -45 | -32 | 9.5 |
| [^18^O. ^18^O] AMPBH_3__1 | 348.0 | 67.0 | -45 | -66 | 9.5 |
| [^18^O. ^18^O] AMPBH_3__2 | 348.0 | 134.1 | -45 | -38 | 9.5 |
| [^18^O. ^18^O] AMPBH_3__3 | 348.0 | 81.0 | -45 | -32 | 9.5 |
| CMP_1 | 322.0 | 79.0 | -55 | -50 | 8.2 |
| CMP_2 | 322.0 | 110.0 | -55 | -50 | 8.2 |
| UMP_1 | 323.0 | 79.0 | -55 | -40 | 8.5 |
| UMP_2 | 323.0 | 111.0 | -55 | -50 | 8.5 |

*Table S2. Fitting parameters of prepared calibration curves and limit of quantification (LOQ)*

| Analite | a | u_a_ | R^2^ | LOQ (μM) |
| --- | --- | --- | --- | --- |
| AMP | 1.087 | 0.0243 | 0.9970 | 0.12 |
| GMP | 1.145 | 0.0253 | 0.9971 | 0.10 |
| AMPS | 1.227 | 0.0065 | 0.9997 | 0.03 |
| AMPH | 2.157 | 0.0289 | 0.9999 | 0.20 |
| AMPBH_3_ | 1.118 | 0.0131 | 0.9992 | 0.22 |

*Table S3. Determined frequencies of phosphorothioate and boranophosphate moieties in polyA tails of RNA G1 and RNA H1 and initial velocities of RNA G1 and RNA H1 deadenylation with CNOT7*

| **ATP:ATPαS D1^[a]^** | **PAP**  **(% of ATPαS D1)^[b]^** | **LC-MS/MS**  **(% of AMPS with SD)^[c]^** | **Initial velocity of polyA tail deadenylation with CNOT7^[d]^** |
| --- | --- | --- | --- |
| 10:0 | 0 | 0 ± 0 | 4.37 ± 0.02 |
| 9:1 | 10 | 6 ± 2 | 1.49 ± 0.03 |
| 4:1 | 20 | 13 ± 5 | 0.99 ± 0.04 |
| 3:1 | 25 | 15 ± 3 | 0.65 ± 0.03 |
| 2:1 | 33 | 23 ± 10 | 0.22 ± 0.02 |
| 1:1 | 50 | 37 ± 9 | n.d. |

| **ATP:ATPαBH_3_ D1^[a]^** | **PAP**  **(% of ATPαBH_3_ D1) ^[b]^** | **LC-MS/MS**  **(% of AMBH_3_ + AMPH with SD)^[c]^** | **Initial velocity of polyA tail deadenylation with CNOT7^[d]^** |
| --- | --- | --- | --- |
| 10:0 | 0 | 0 ± 0 | 4.13 ± 0.22 |
| 14:1 | 6.66 | 5 ± 4 | n.d. |
| 9:1 | 10 | 6 ± 4 | 4.11 ± 0.08 |
| 5:1 | 20 | 6 ± 3 | 3.47 ± 0.12 |
| 3:1 | 25 | 11 ± 10 | 3.22 ± 0.14 |
| 1:1 | 50 | 14 ± 5 | 2.57 ± 0.13 |
| 0:10 | 100 | 45 ± 24 | 1.86 ± 0.17 |

^[a]^ proportion of ATP to phosphate-modified ATP analogue (ATPαS D1 or ATPαBH_3_ D1) in PAP reaction mix; ^[b]^ percentage of phosphate-modified ATP analogue in PAP reaction mix; ^[c]^ percentage of phosphate-modified ATP analogue determined by LC-MS/MS (products of mRNA degradation with SVPDE and CNOT7 are presented in brackets); ^[d]^ initial velocity of RNA G1 and RNA H1 deadenylation with CNOT7. The initial velocity was calculated as a number of nucleotides removed from the 3’ end of polyA by CNOT7 deadenylase during the reaction period (nt/min), including data points at the linear reaction phase (0-40 min for unmodified polyA tails and for boranophosphate-modified polyA tails; 0-120 min for phosphorothioate-modified polyA tails). Image Lab Software (BioRad) was used for estimation of mRNA lengths. Data shown are means ± SD of three independent experiments for three independent biological replicates (two replicates in case of 0:10 variant); n.d. – not determined

*Table S4. Estimation of polyA tails lengths by analysis of mRNA migration in agarose gel and by LC-MS/MS method*

| **ATP:ATPαS D1^[a]^** | **Dominant length of polyA tail (gel)**  **(nt)^[b]^** | **Average length of polyA tail (LC-MS/MS)**  **(nt)^[c]^** |
| --- | --- | --- |
| 10:0 | 223 ± 20 | 165 ± 30 |
| 9:1 | 190 ± 25 | 142 ± 28 |
| 4:1 | 262 ± 57 | 183 ± 75 |
| 3:1 | 276 ± 67 | 221 ± 78 |
| 2:1 | 338 ± 70 | 257 ± 106 |
| 1:1 | n.d. | 313 ± 79 |

| **ATP:ATPαBH_3_ D1^[a]^** | **Dominant length of polyA tail (gel)**  **(nt)^[b]^** | **Average length of polyA (LC-MS/MS)**  **(nt)^[c]^** |
| --- | --- | --- |
| 10:0 | 191 ± 48 | 119 ± 46 |
| 14:1 | 197 ± 45 | 107 ± 40 |
| 9:1 | 207 ± 48 | 106 ± 29 |
| 5:1 | 192 ± 74 | 127 ± 48 |
| 3:1 | 170 ± 72 | 88 ± 47 |
| 1:1 | 134 ± 122 | 79 ± 48 |
| 0:10 | 156 ± 26 | 53 ± 36 |

^[a]^ proportion of ATP to phosphate-modified ATP analogue (ATPαS D1 or ATPαBH_3_ D1) in PAP reaction mix; ^[b]^ polyA tail length estimated using Image Lab Software for dominant bands of mRNA separated on 1% 1×TBE agarose gel. Data shown are means ± SD of three independent biological replicates; ^[c]^ polyA tail length determined by LC-MS/MS. Data shown are means ± SD of three independent experiments for three independent biological replicates; n.d. – not determined due to sample heterogeneity

Table S5. Initial velocities of unmodified and modified RNA G1 deadenylation with CNOT7

|  | **ATP:ATPαS D1^[a]^** | **V_0_ (nt/min)^[b]^** |
| --- | --- | --- |
| **1^st^** | 10:0 | 4.7 ± 0.14 |
|  | 9:1 | 1.63 ± 0.06 |
|  | 4:1 | 0.95 ± 0.09 |
|  | 3:1 | 0.63 ± 0.03 |
|  | 2:1 | 0.32 ± 0.02 |
| **2^nd^** | 10:0 | 4.47 ± 0.19 |
|  | 9:1 | 1.37 ± 0.05 |
|  | 4:1 | 0.95 ± 0.04 |
|  | 3:1 | 0.6 ± 0.05 |
|  | 2:1 | 0.19 ± 0.04 |
| **3^rd^** | 10:0 | 4.05 ± 0.14 |
|  | 9:1 | 1.56 ± 0.09 |
|  | 4:1 | 0.92 ± 0.04 |
|  | 3:1 | 0.74 ± 0.02 |
|  | 2:1 | 0.16 ± 0.01 |
| **Average^[c]^** | 10:0 | 4.37 ± 0.02 |
|  | 9:1 | 1.49 ± 0.03 |
|  | 4:1 | 0.99 ± 0.04 |
|  | 3:1 | 0.65 ± 0.03 |
|  | 2:1 | 0.22 ± 0.02 |
| **Relative V_0_^[d]^** | 10:0 | 1 ± 0.47 |
|  | 9:1 | 0.34 ± 0.01 |
|  | 4:1 | 0.23 ± 0.01 |
|  | 3:1 | 0.15 ± 0.01 |
|  | 2:1 | 0.05 ± 0.01 |

^[a]^ proportion of ATP:ATPαS D1 used as a substrate for PAP in polyadenylation reaction; ^[b]^ initial velocity of deadenylation with CNOT7 of RNA G1 variants (unmodified (10:0) and modified (9:1, 4:1, 3:1 and 2:1)). The initial velocity was calculated as a number of nucleotides removed from the 3’ end of polyA by CNOT7 deadenylase during the reaction period (nt/min), including data points at the linear reaction phase (0-40 min for unmodified polyA (10:0) and 0-120 min for modified polyA (9:1, 4:1, 3:1 and 2:1)). Image Lab Software (BioRad) was used for estimation of mRNA length. Data shown are means ± SE of three independent experiments for three independent biological replicates; ^[c]^ average V_0_ calculated for three independent biological replicates ± SD; ^[d]^ V_0_ normalized to the values obtained for unmodified polyA tails in three independent biological replicates

*Table S6. Initial velocities of unmodified and modified RNA H1 deadenylation with CNOT7*

|  | **ATP:ATPαBH_3_ D1^[a]^** | **V_0_ (nt/min)^[b]^** |
| --- | --- | --- |
| **1^st^** | 10:0 | 4.75 ± 0.26 |
|  | 9:1 | 4.25 ± 0.14 |
|  | 5:1 | 3.45 ± 0.09 |
|  | 3:1 | 3.32 ± 0.07 |
|  | 1:1 | 3.17 ± 0.19 |
|  | 0:10 | 1.86 ± 0.14 |
| **2^nd^** | 10:0 | 4.17 ± 0.22 |
|  | 9:1 | 4.55 ± 0.03 |
|  | 5:1 | 3.9 ± 0.11 |
|  | 3:1 | 3.32 ± 0.1 |
|  | 1:1 | 2.52 ± 0.07 |
|  | 0:10 | 1.6 ± 0.07 |
| **3^rd^** | 10:0 | 3.47 ± 0.19 |
|  | 9:1 | 3.52 ± 0.07 |
|  | 5:1 | 3.07 ± 0.16 |
|  | 3:1 | 3 ± 0.26 |
|  | 1:1 | 2 ± 0.14 |
| **Average**^[c]^ | 10:0 | 4.13 ± 0.22 |
|  | 9:1 | 4.11 ± 0.08 |
|  | 5:1 | 3.47 ± 0.12 |
|  | 3:1 | 3.22 ± 0.14 |
|  | 1:1 | 2.57 ± 0.13 |
|  | 0:10 | 1.86 ± 0.17 |
| **Relative V_0_**^[d]^ | 10:0 | 1 ± 0.05 |
|  | 9:1 | 0.99 ± 0.02 |
|  | 5:1 | 0.84 ± 0.03 |
|  | 3:1 | 0.78 ± 0.03 |
|  | 1:1 | 0.62 ±0.03 |
|  | 0:10 | 0.45 ± 0.04 |

^[a]^ proportion of ATP:ATPαBH_3_ D1 used as a substrate for PAP in polyadenylation reaction; ^[b]^ initial velocity of deadenylation with CNOT7 of RNA H1 variants (unmodified (10:0) and modified (9:1, 5:1, 3:1, 1:1 and 0:10)). The initial velocity was calculated as a number of nucleotides removed from the 3’ end of polyadenylated RNA H1 by CNOT7 deadenylase during the reaction period (nt/min), including data points at the linear reaction phase (0-40 min for all RNA H1). Image Lab Software (BioRad) was used for estimation of mRNA length. Data shown are means ± SE of three independent experiments for three independent biological replicates; ^[c]^ average V_0_ calculated for three independent biological replicates (two replicates in case of 0:10 variant) ± SD; ^[d]^ V_0_ normalized to the values obtained for unmodified polyA tails in three independent biological replicates (two replicates in case of 0:10 variant)

*Table S7. Chromatograms and spectra of compounds used in LC-MS/MS method development and in in vitro transcription*

| **AMP** |
| --- |
| 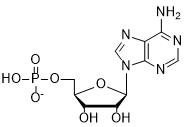 |
| **HPLC** |
|  |
| **MS** |
| **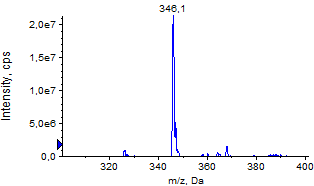** |
| **MS/MS** |
| **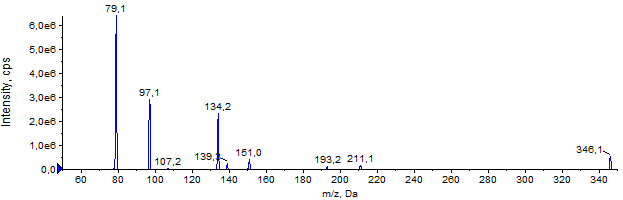** |
| **MRM** |
|  |

| **[^18^O, ^18^O]AMP** |
| --- |
| 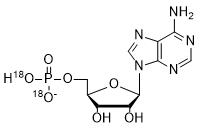 |
| **HPLC** |
|  |
| **MS** |
| 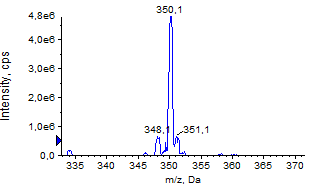 |
| **MS/MS** |
| 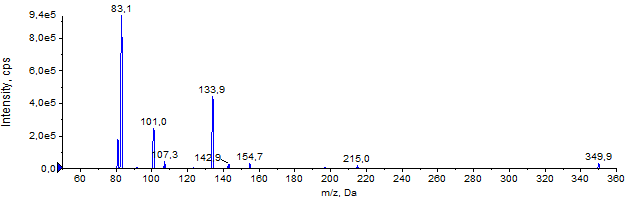 |
| **MRM** |
|  |

| **AMPS** |
| --- |
| 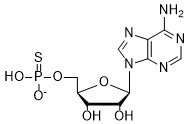 |
| **HPLC** |
| **** |
| **MS** |
| 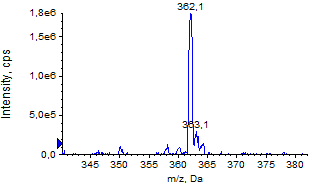 |
| **MS/MS** |
| 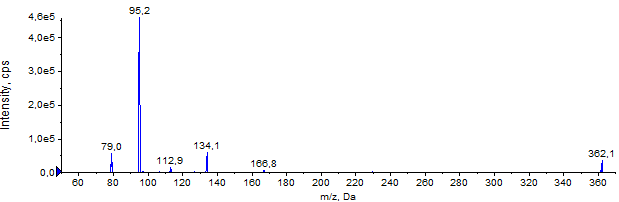 |
| **MRM** |
|  |

| **[^18^O, ^18^O]AMPS** |
| --- |
| 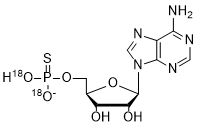 |
| **HPLC** |
|  |
| **MS** |
| **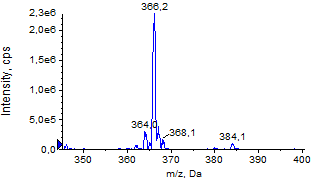** |
| **MS/MS** |
| **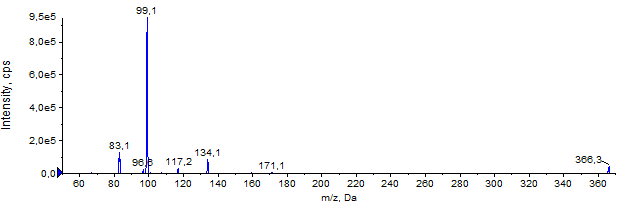** |
| **MRM** |
|  |

| **GMP** |
| --- |
| 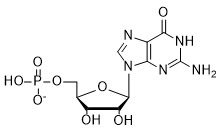 |
| **HPLC** |
|  |
| **MS** |
| 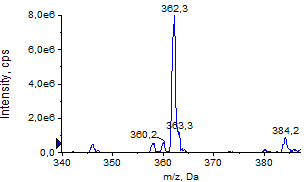 |
| **MS/MS** |
| 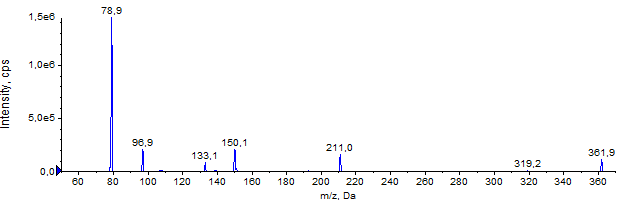 |
| **MRM** |
|  |

| **[^18^O, ^18^O]GMP** |
| --- |
| 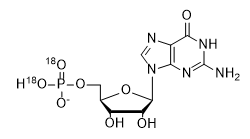 |
| **HPLC** |
|  |
| **MS** |
| **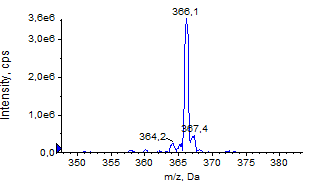** |
| **MS/MS** |
| 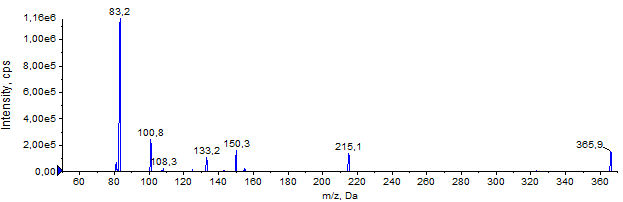 |
| **MRM** |
|  |

| **AMPH** |
| --- |
| 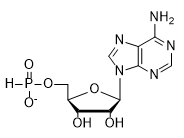 |
| **HPLC** |
|  |
| **MS** |
| 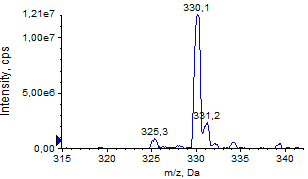 |
| **MS/MS** |
| 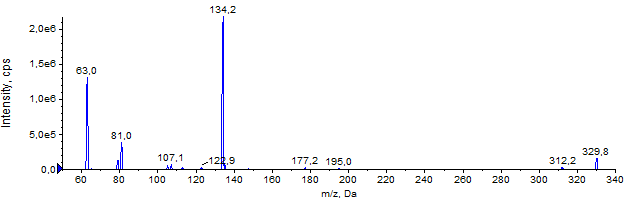 |
| **MRM** |
|  |

| **[^18^O, ^18^O]AMPH** |
| --- |
| 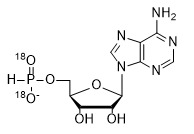 |
| **HPLC** |
|  |
| **MS** |
| 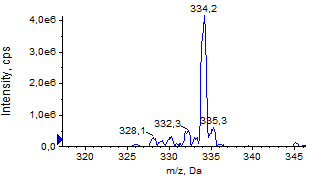 |
| **MS/MS** |
| 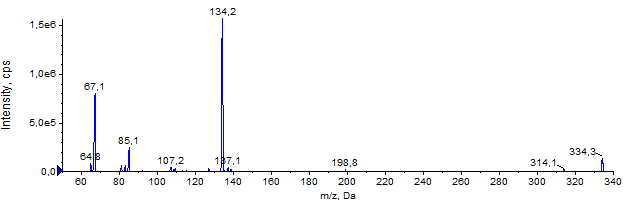 |
| **MRM** |
|  |

| **UMP** |
| --- |
| 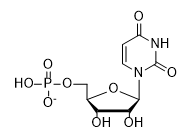 |
| **MRM** |
|  |

| **CMP** |
| --- |
| 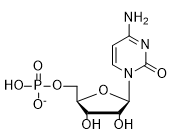 |
| **MRM** |
|  |

| **AMPBH_3_** |
| --- |
| 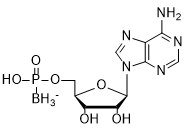 |
| **HPLC** |
|  |
| **MS** |
| 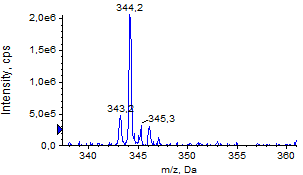 |

| **[^18^O, ^18^O] AMPBH_3_** |
| --- |
| 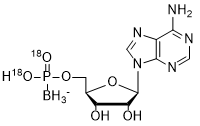 |
| **HPLC** |
|  |
| **MS** |
| 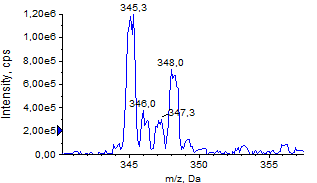 |

| **[D]ATP** |
| --- |
| 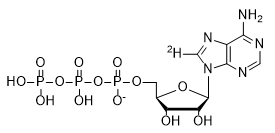 |
| **HPLC** |
|  |
| **MS** |
| 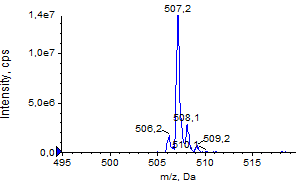 |

# Supplementary Figures


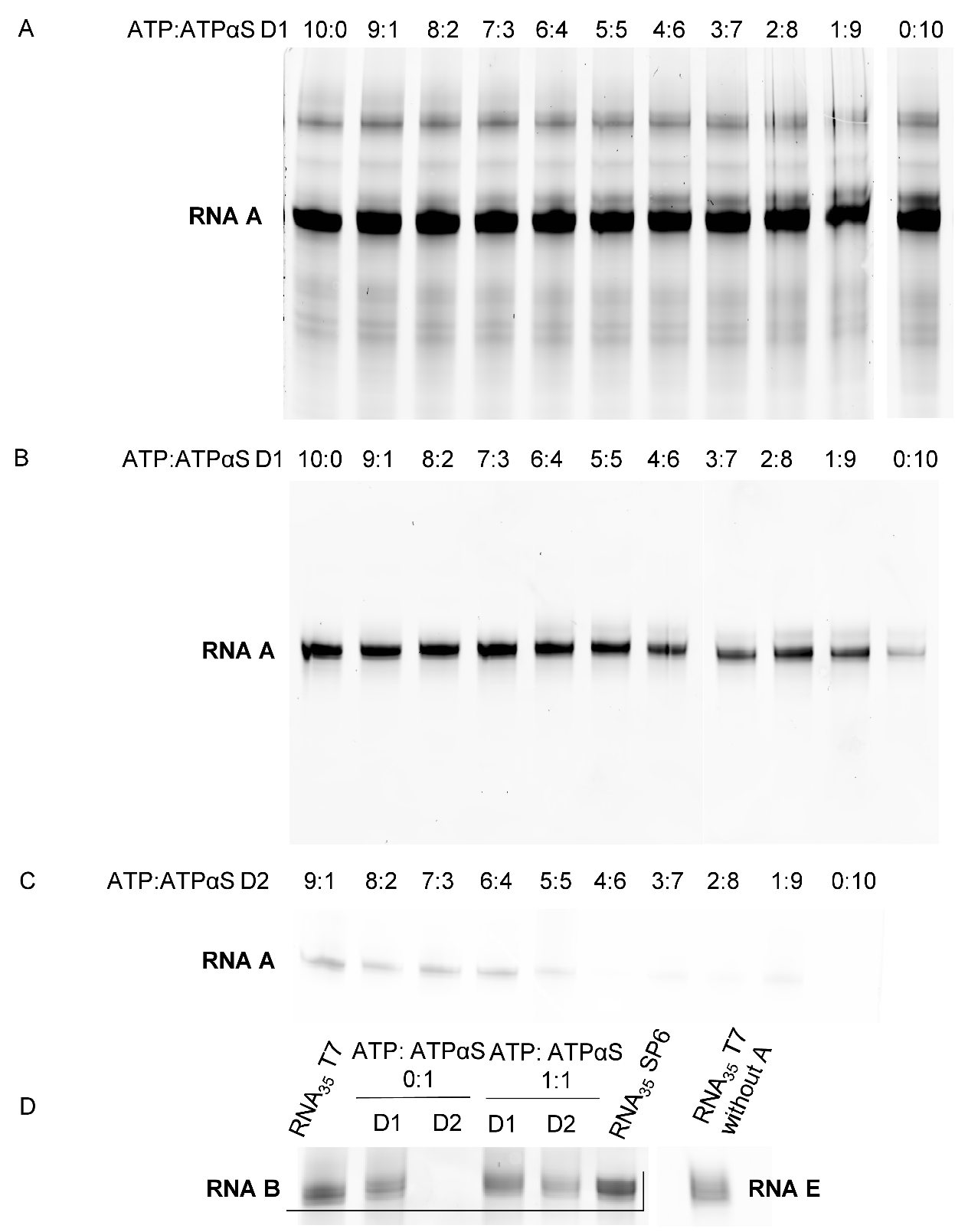


*Figure S1. Analysis of short RNAs obtained by in vitro transcription using T7 and SP6 polymerase. In vitro transcribed (IVT) RNAs were obtained by a standard protocol: RNA Pol buffer (40 mM Tris-HCl pH 7.9, 6 mM MgCl_2_, 1 mM DTT, 2 mM spermidine), 10 U/µL T7 or 1 U/µL SP6 RNA polymerase (ThermoFisher Scientific), 1 U/µL RiboLock RNase Inhibitor (ThermoFisher Scientific), 0.5 mM CTP/UTP/GTP and 0.5 mM different ATP:ATPαS ratios, and 0.4 µM annealed oligonucleotides as a template. Procedure for RNA without A was different (see Material and Methods),* ATPαS D1 is *S*_P_-ATPαS isomer, ATPαS D2 is *R*_P_-ATPαS isomer*. (A) IVT RNAs using SP6 polymerase and different ratios of ATP:ATPαS D1. The crude IVT RNAs were purified using RNA Clean & Concentrator-25 (Zymo Research). (B) Gel-purified samples using PAA elution buffer (0.3 M sodium acetate, 1 mM EDTA, 0.05% Triton X-100), precipitated with isopropanol and dissolved in water. (C) IVT RNAs using SP6 polymerase and different ratios of ATP:ATPαS D2. The crude RNAs were purified using RNA Clean & Concentrator-25 (Zymo Research) and gel-purified. (D) IVT RNAs using T7 polymerase and different ratios of ATP:ATPαS D1 or D2. The crude IVT RNAs were purified using RNA Clean & Concentrator-25 (Zymo Research) and gel-purified. RNA without A in sequence were purified using HPLC method described in Material and Methods.*


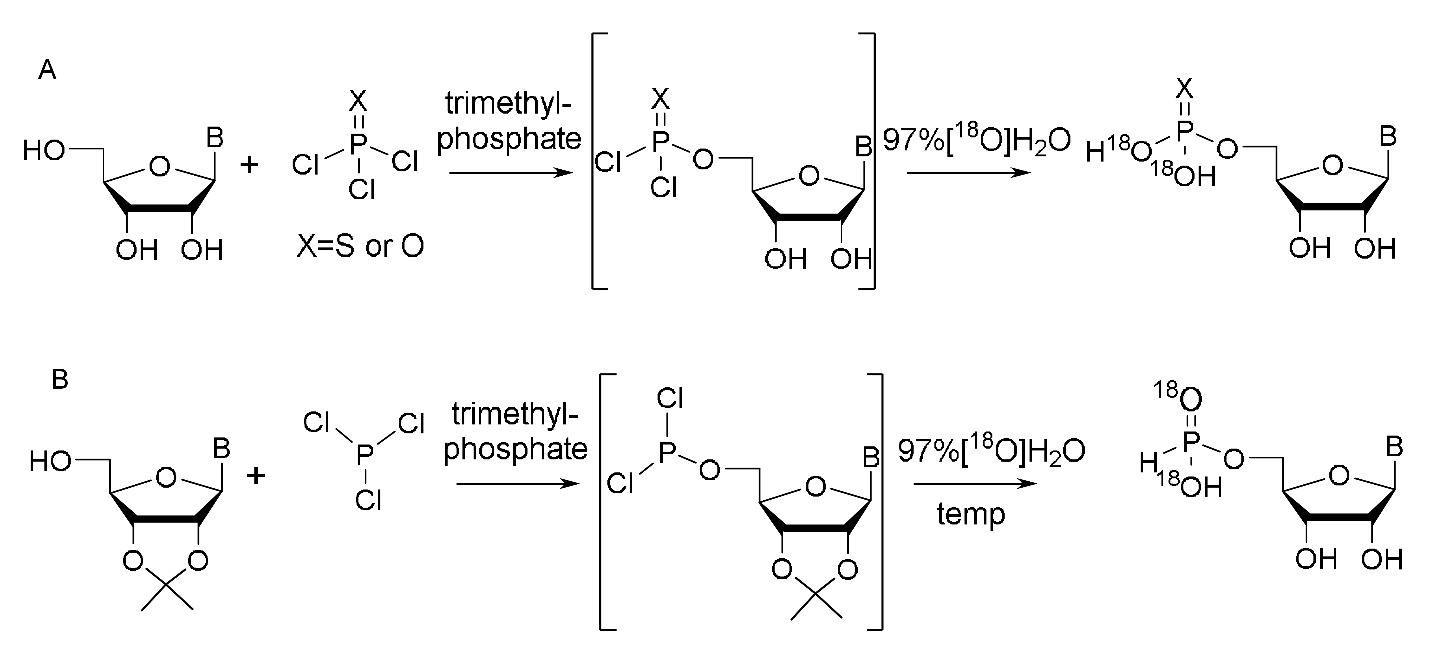


Figure S2. Syntheses of nucleotides and their isotopically labelled analogues carried out in this work: (A) - Nucleoside 5’-phosphorylation and 5’-thiophosphorylation, (B) 5’- phosphitylation of 2’,3’-protected nucleosides.

Figure S3. Calibration curves for analyzed compounds.


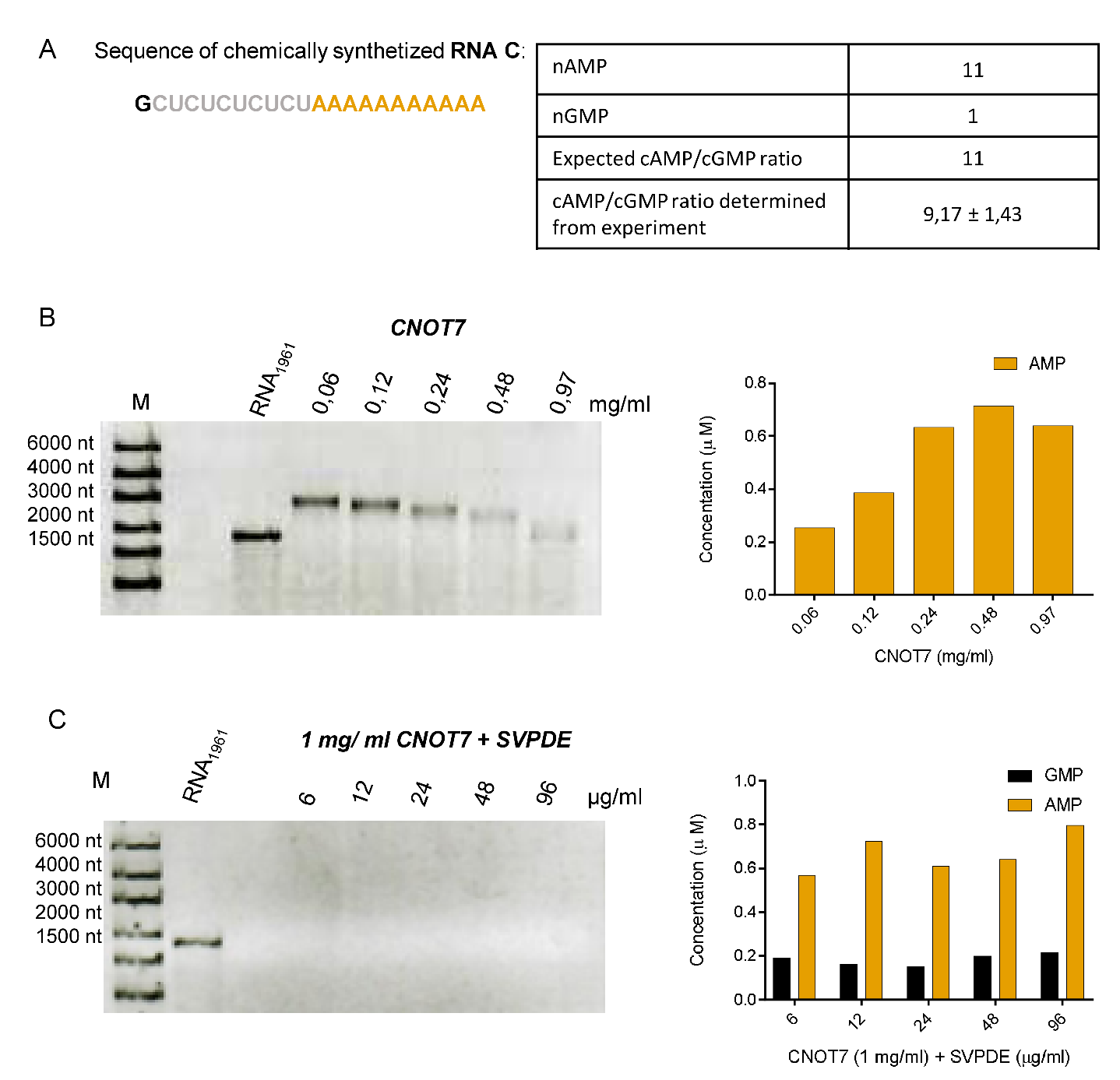


*Figure S4. Optimization of degradation conditions. Typical RNA degradation reaction (20 µL) was incubated at 37 °C for 1 h and contained: 40 ng RNA, buffer (5 mM MgCl_2_, 10 mM Tris, pH 8.0, 50 mM KCl, 10 mM DTT) and appropriate amount of SVPDE and/or CNOT7 enzyme. (A) Preliminary method validation with synthetic unmodified RNA (RNA C), (B) optimization of CNOT7 enzyme for digestion mRNA, (C) optimization amount of SVPDE for mRNA degradation using established amount of CNOT7.*


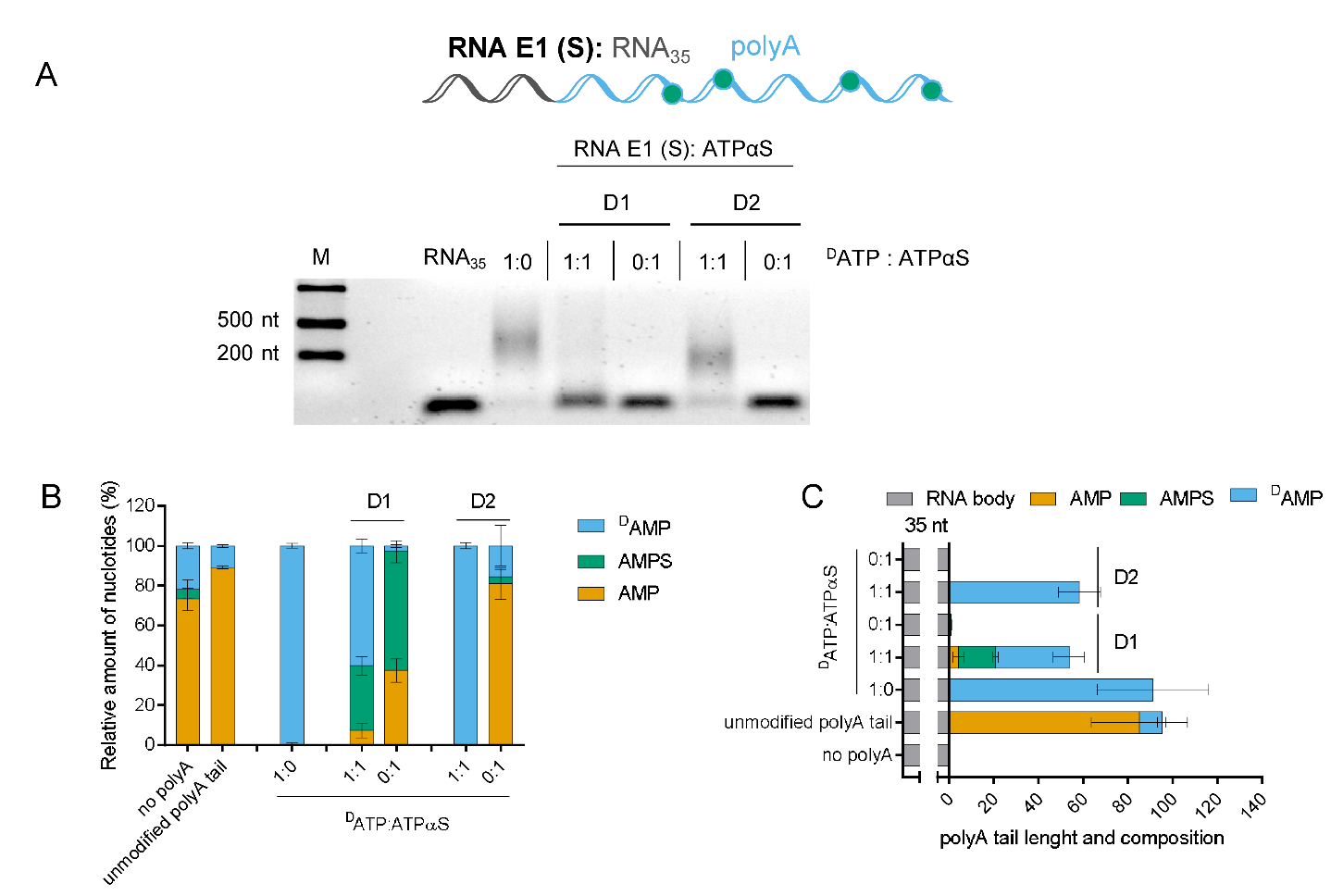


Figure S5. Analysis of short RNAs (RNA E1) (with U instead of A in the sequence) with polyA tails added by PAP polymerase using different ^D^ATP:ATP analogue ratio. Obtained ^D^ATP included 10% of ATP, which was taken into account during data analysis. RNA E1 were digested using SVPDE and CNOT7 enzymes in 1h at 37 ^O^C (see Materials and Methods for further details). (A) Data shown come from a single agarose gel. (B) Amount of nucleotides after RNA decompositions determined based on LC-MS/MS quantification analysis, data normalized to GMP. (C) PolyA tail length was calculated according procedure described in Materials and Methods.


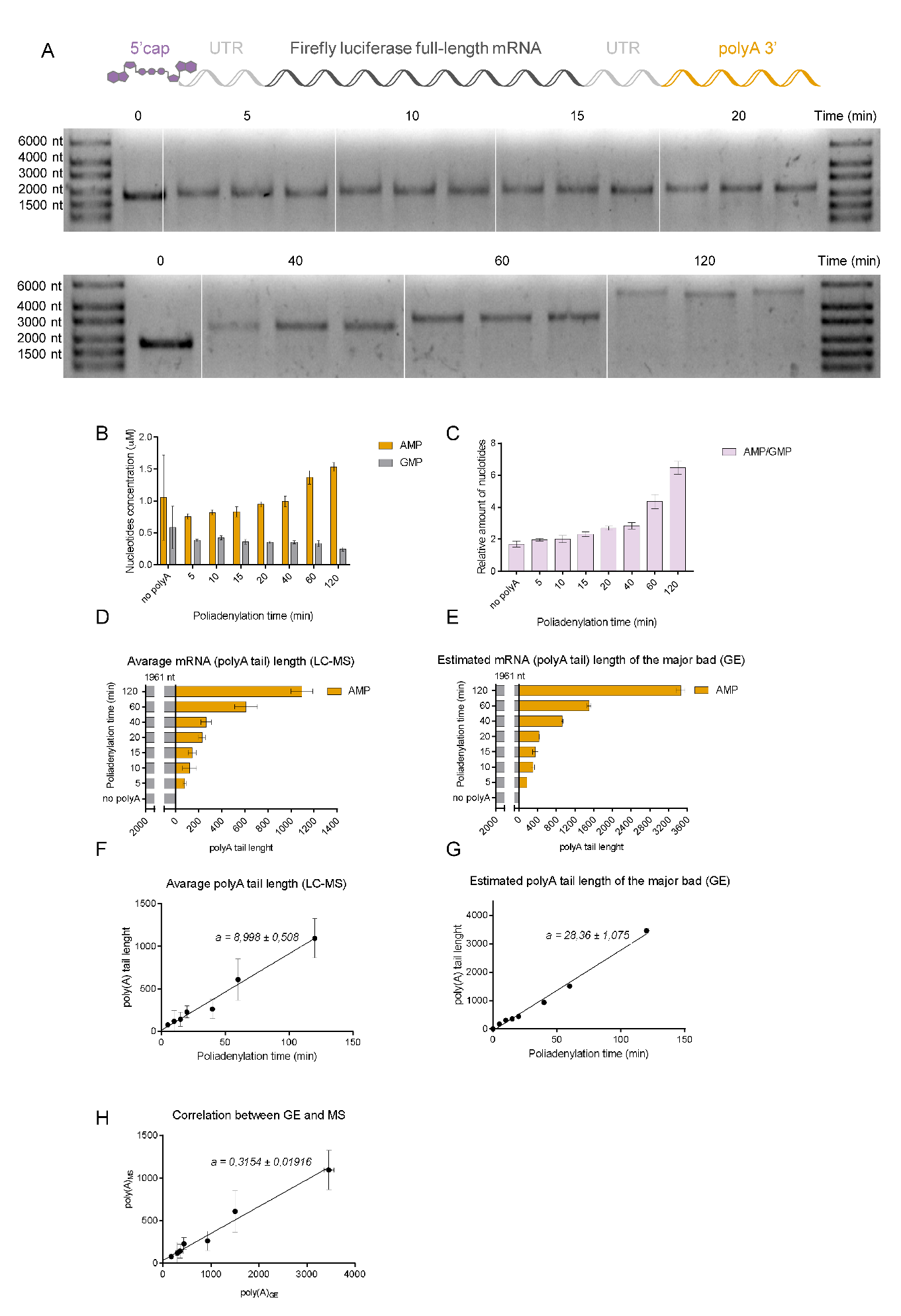


Figure S6. Preparation of Firefly luciferase with unmodified polyA tails with various lengths. (A) Firefly luciferase without polyA tail (RNA F1) was in vitro transcribed (IVT) with SP6 RNA polymerase using unmodified NTPs and co-transcriptionally 5’-capped with ARCA cap analogue. The crude IVT mRNA was purified with NucleoSpin RNA Clean-up XS (Macherey-Nagel), and used as a primer for polyA polymerase (PAP). Polyadenylation reaction mixture (10 μL) contained: 250 ng RNA F1, 0.1 U/μL PAP, 0.5 mM ATP and 20 U RiboLock RNase inhibitor. RNA F1 was incubated with PAP for 0-120 min, and the reaction was stopped with EDTA (f.c. 25 mM). Polyadenylated mRNAs (RNA F2) were purified with NucleoSpin RNA Clean-up XS. The quality and length of polyadenylated mRNAs were analyzed by loading the same amount of mRNA (50 ng) on 1% 1×TBE agarose gel. Samples were prepared in three independent replicates. (B) Data obtained from LC-MS analysis. Concentrations of GMP and AMP after RNA decomposition. (C) AMP:GMP ratio is higher due to polyA tail elongation, (D) mRNA length obtained from LC-MS analysis and electrophoresis gel separation (E), (F) PolyA tail length obtained from LC-MS analysis and electrophoresis gel separation dependent on polyadenylation time (G), (H) correlation between GE and MS.


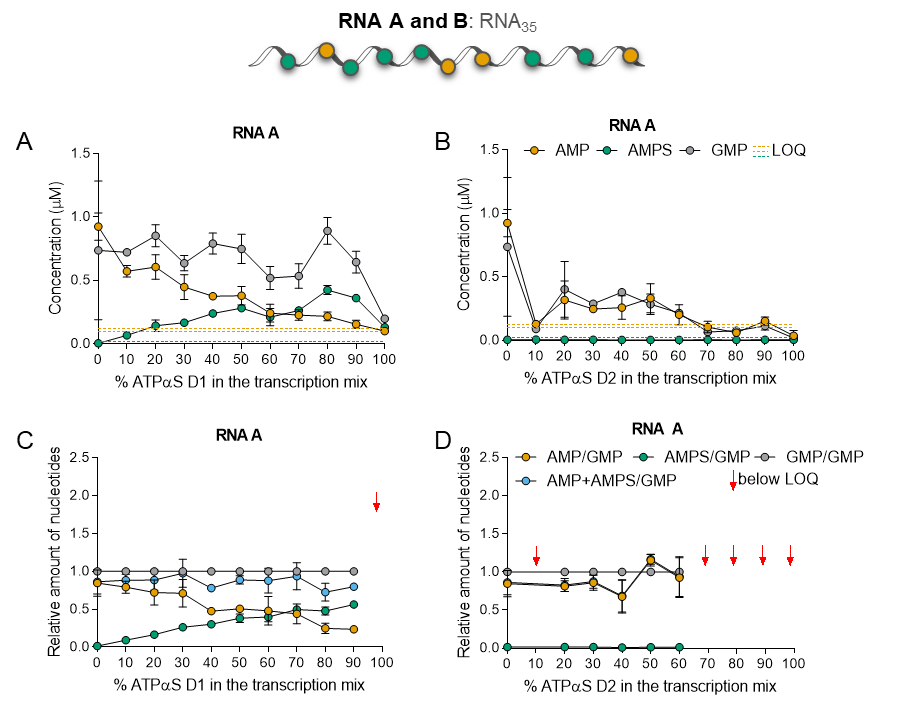


*Figure S7. SP6 and T7 polymerases accept ATPαS D1 as a substrate. Short RNAs (RNA A or B) were obtained by in vitro transcription by SP6 or T7 RNA polymerase, respectively, in the presence of different ATP:ATPαS D1/D2 ratios. In vitro transcribed (IVT) RNAs (20 ng) were incubated with 3 µg/mL snake venom phosphodiesterase (SVPDE) for 1 h at 37 °C (see Materials and Methods for further details). Concentrations of nucleotides after RNA digestion are determined based on liquid chromatography–tandem mass spectrometry (LC-MS/MS) analysis (A, B). The data are normalized to guanosine monophosphate (GMP) to account for differences in transcript amounts that were analyzed (C, D). Data points represent mean values from triplicate experiments ± standard deviation (SD).*


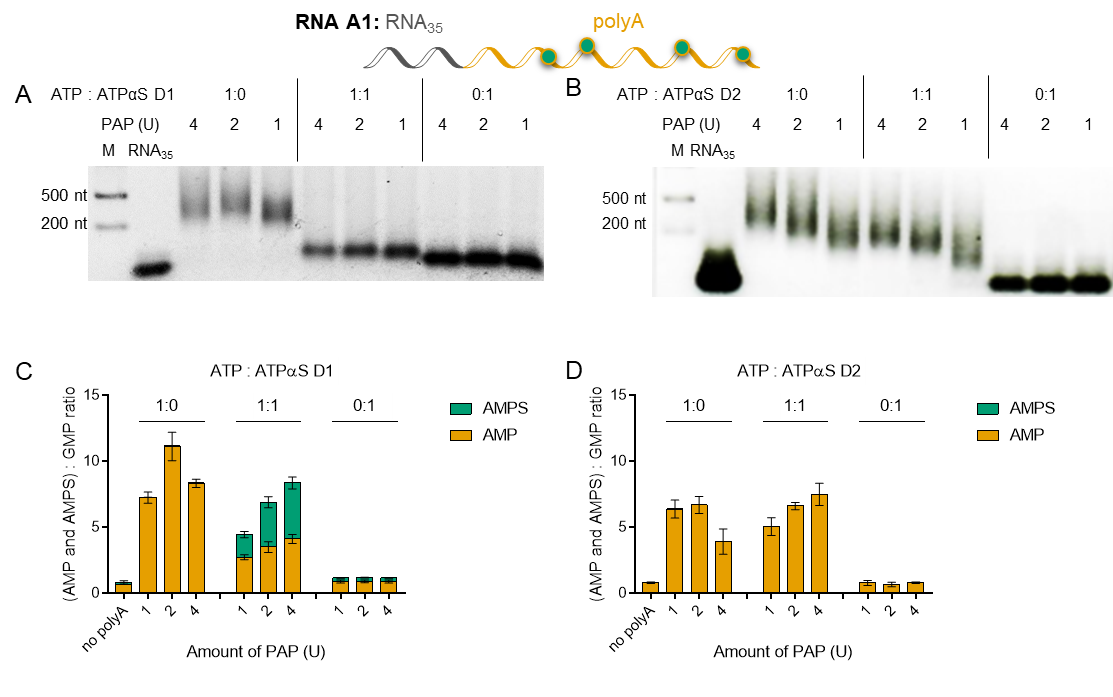


*Figure S8. PolyA polymerase (PAP) incorporates ATPαS D1 in the presence of ATP to produce modified polyA tails. Analysis of short RNAs with polyA (RNA A1) tails added by PAP using different ATP:ATPαS ratios (1 mM). Short RNAs (RNA A) are obtained by SP6 polymerase. RNA A1 (20 ng) is digested using the snake venom phosphodiesterase (SVPDE) and Ccr4-Not transcription complex subunit 7 (CNOT7) enzymes for 1 h at 37 ^°^C (see Materials and Methods for further details). Data points represent mean values from triplicate experiments ± standard error of the mean (SEM). (A, C) data obtained for ATPαS D1, (B, D) data obtained for ATPαS D2. Data shown are from a single agarose gel (A, B). RNA composition is determined based on liquid chromatography–tandem mass spectrometry (LC-MS/MS) quantification analysis followed by normalization to guanosine monophosphate (GMP) (C, D).*


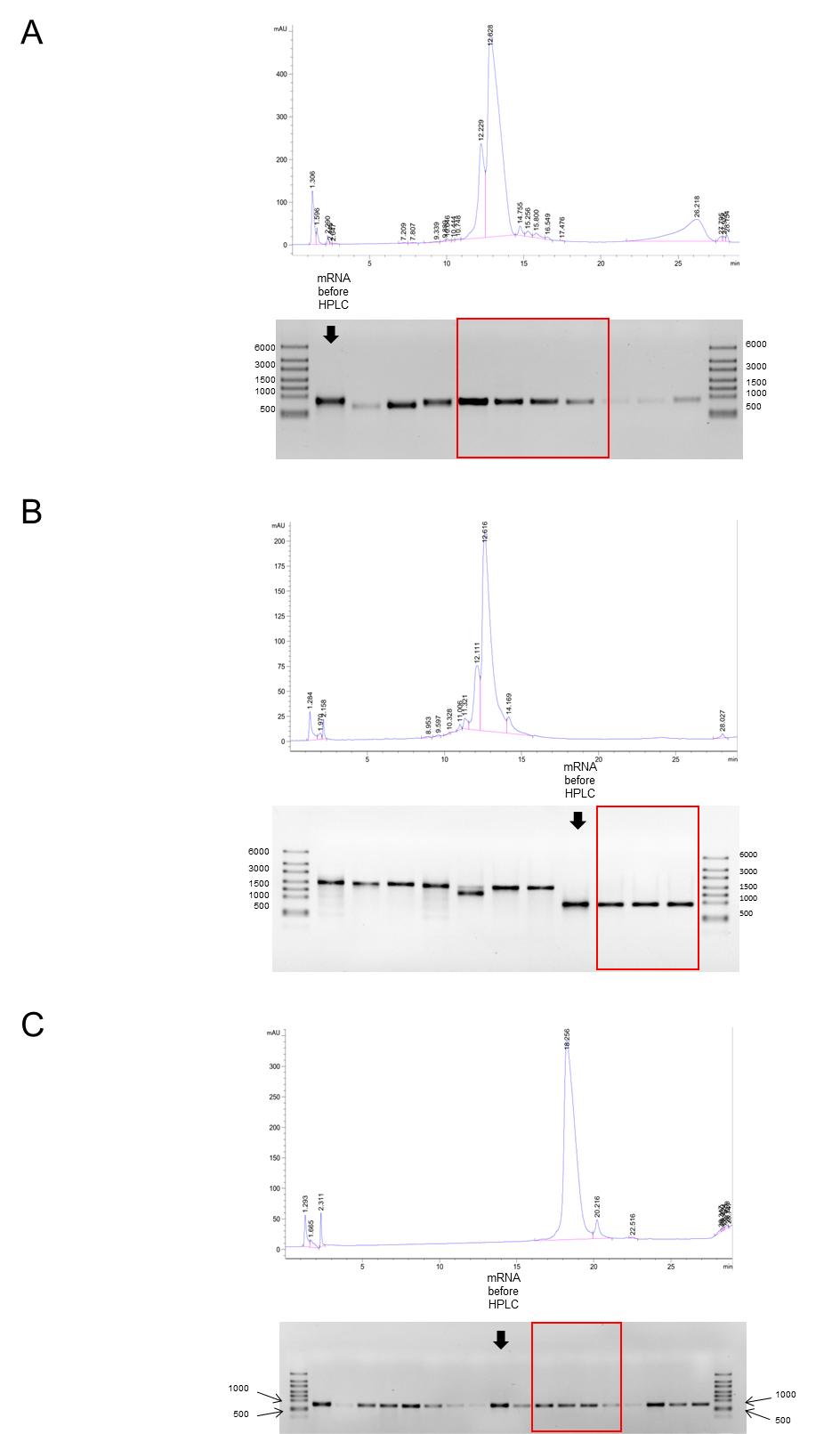


*Figure S9. In vitro transcription and purification of all types of mRNA encoding Gaussia luciferase used for analysis of composition, translational properties and stability. IVT mRNAs without polyA tail were obtained by standard protocol (see Materials and Methods) using SP6 RNA polymerase, unmodified NTPs and GTP:ARCA 1:10 ratio for co-transcriptional mRNA capping. The crude IVT mRNAs were initially purified with NucleoSpin RNA Clean-up XS (Macherey-Nagel), followed by purification on Agilent Technologies Series 1200 HPLC using RNASep™ Prep – RNA Purification Column (ADS Biotec). Collected fractions were precipitated with 0.3 M sodium acetate pH 5.2 and isopropanol at RT, resuspend in water, and analyzed on 1% 1×TBE agarose gel. mRNA from selected fractions (marked with a red frame) were pooled together and used as a primer for PAP. (A) Elution profile and agarose gel analysis for IVT RNA G, (B) RNA I, (C) and for RNA H.*


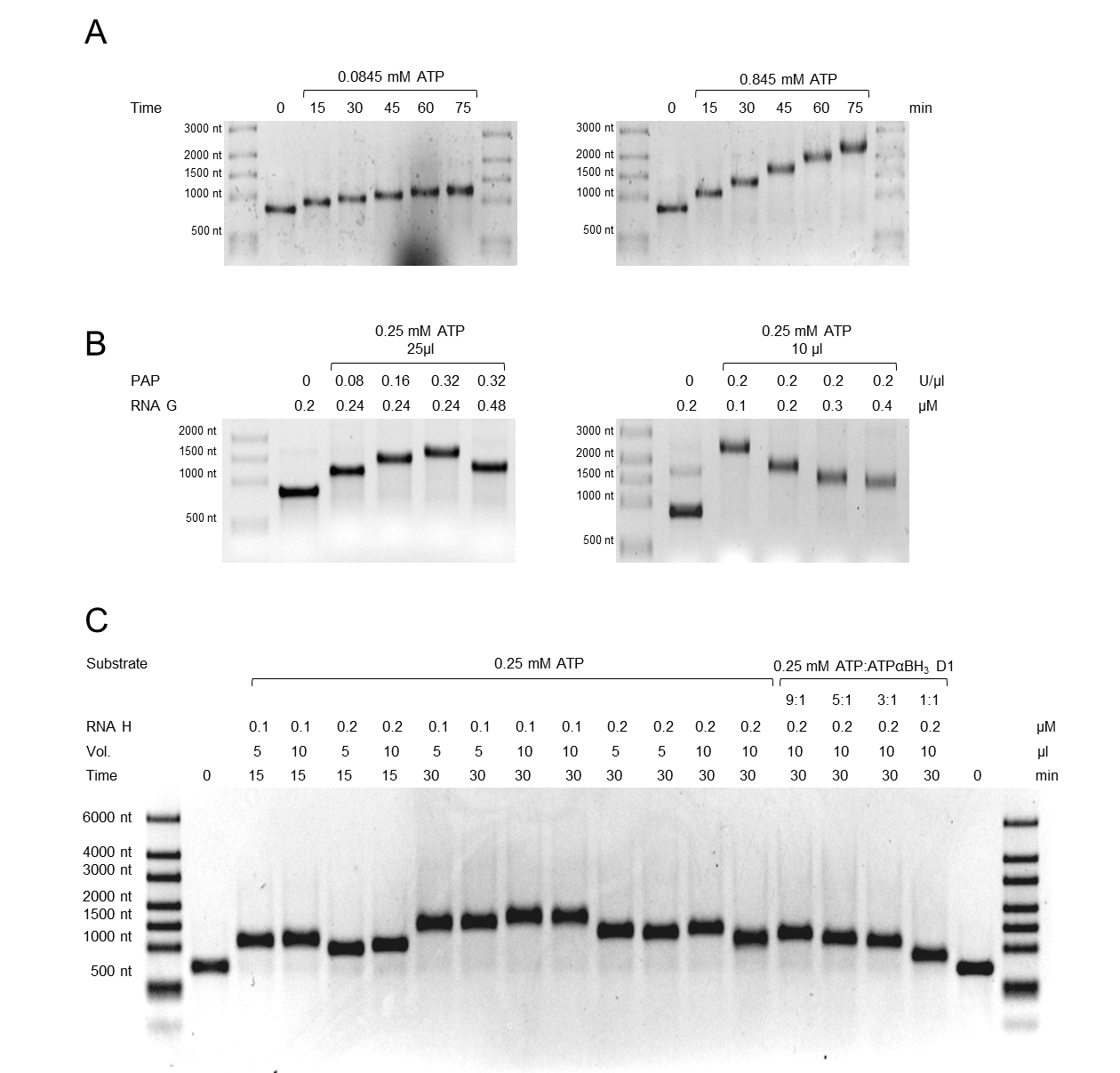


*Figure S10. Optimization of mRNA polyadenylation by PAP. (A) The time-course of Gaussia mRNA (RNA G) polyadenylation in a presence of 0.0845 or 0.845 mM ATP. RNA G after in vitro transcription with SP6 RNA polymerase was silica-membrane purified (NucleoSpin RNA Clean-up XS, Macherey-Nagel), and 0.147 μM of RNA G was used as a primer for 0.1 U/μl PAP. At indicated time points equal volume of samples were collected from single reaction mixture incubated at 37 °C and analyzed on 1% 1×TBE agarose gel. (B) Influence of mRNA and PAP concentration on polyA tail extension efficiency. Silica-membrane purified RNA G was incubated with PAP and 0.25 mM ATP for 60’ at 37 °C. Polyadenylation efficiency was tested for variable concentrations of PAP versus RNA G (left panel) and for 0.1-0.4 μM RNA G with unchanged the rest of reaction parameters (right panel). (C) Optimization of polyadenylation conditions for HPLC purified Gaussia mRNA (RNA H). In vitro transcription of RNA H was performed with SP6 RNA polymerase by the standard protocol. IVT RNA H after silica-membrane and HPLC purification was used as a primer for PAP. RNA H was polyadenylated by PAP (0.2-0.4 U/μL) in the presence of 0.25 mM ATP or 0.25 mM of ATP:ATPαBH_3_ D1 at 9:1, 5:1, 3:1 or 1:1 molar ratio (lanes 15-18) and various RNA H concentration (0.1-0.2 μM), reaction volume (5-10 μl) and incubation time (15-30 min). Polyadenylation reaction was stopped with 25 mM EDTA and the lengths of polyadenylated mRNAs were analyzed on 1% 1×TBE agarose gel by loading equal volume of the samples.*


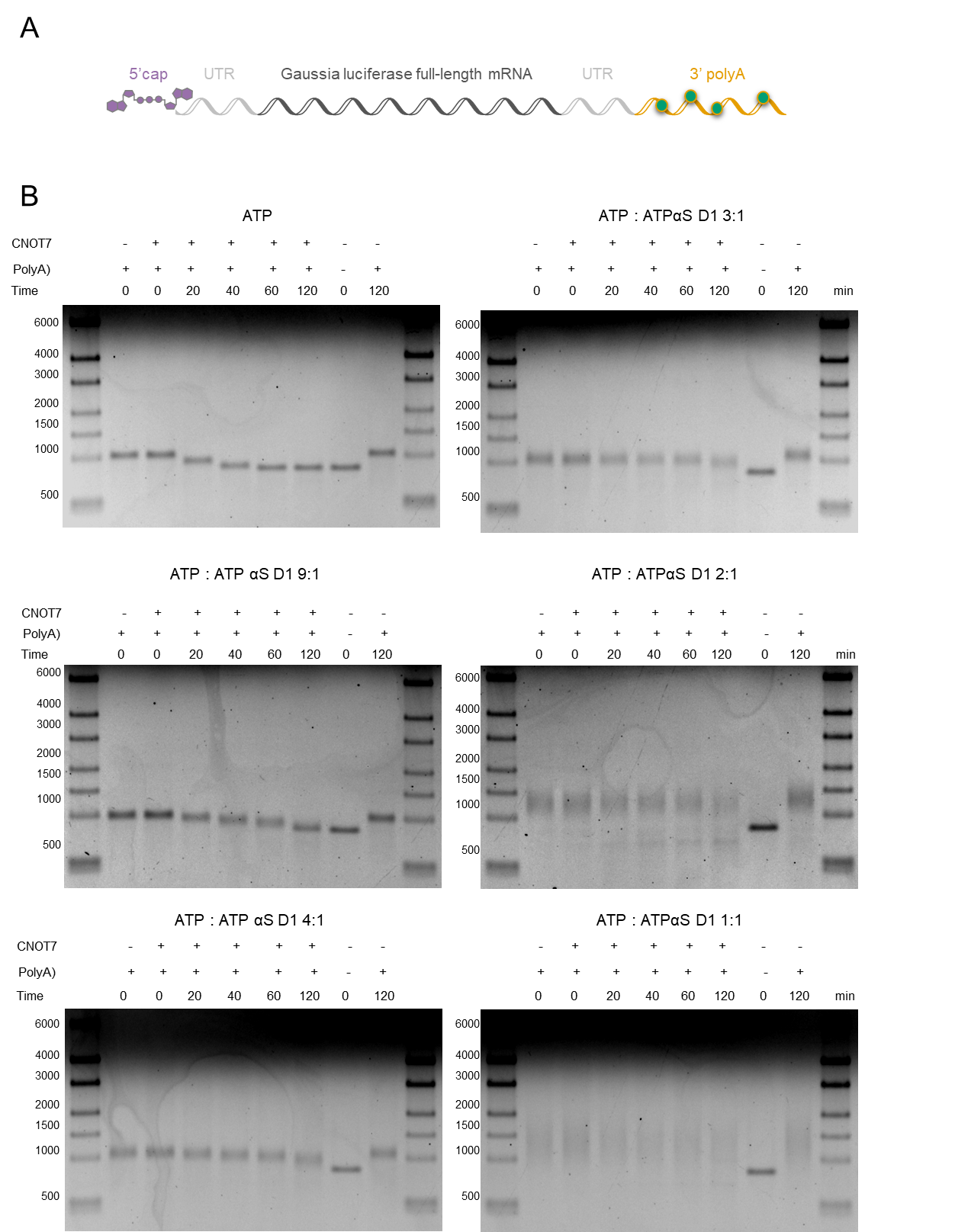


*Figure S11. Analysis of RNA G1 susceptibility to deadenylation. (A) Schematic representation of Gaussia luciferase mRNA (RNA G1) used for degradation assay with human CNOT7 deadenylase. (B) Second repetition of stability analysis with HPLC purified RNA G polyadenylated by PAP and ATP or the mix of ATP:ATPαS D1 at 9:1, 4:1, 3:1, 2:1 or 1:1 molar ratio. RNA G1 containing unmodified polyA tail (marked as ATP or 10:0) served as a positive control of deadenylation. All variants of RNA G1 were incubated with CNOT7 deadenylase for 0-120 min. The reaction was stopped by adding equal volume of loading dye containing 50 mM EDTA, and the polyA tail degradation rate was analyzed on 1% 1×TBE agarose gel stained with ethidium bromide. The lengths of digested polyA tails were estimated using Image Lab 6.0.1 Software (Bio-Rad). The polyA tails degradation rates were estimated as a number of nucleosides removed by CNOT7 deadenylase during the reaction period.*


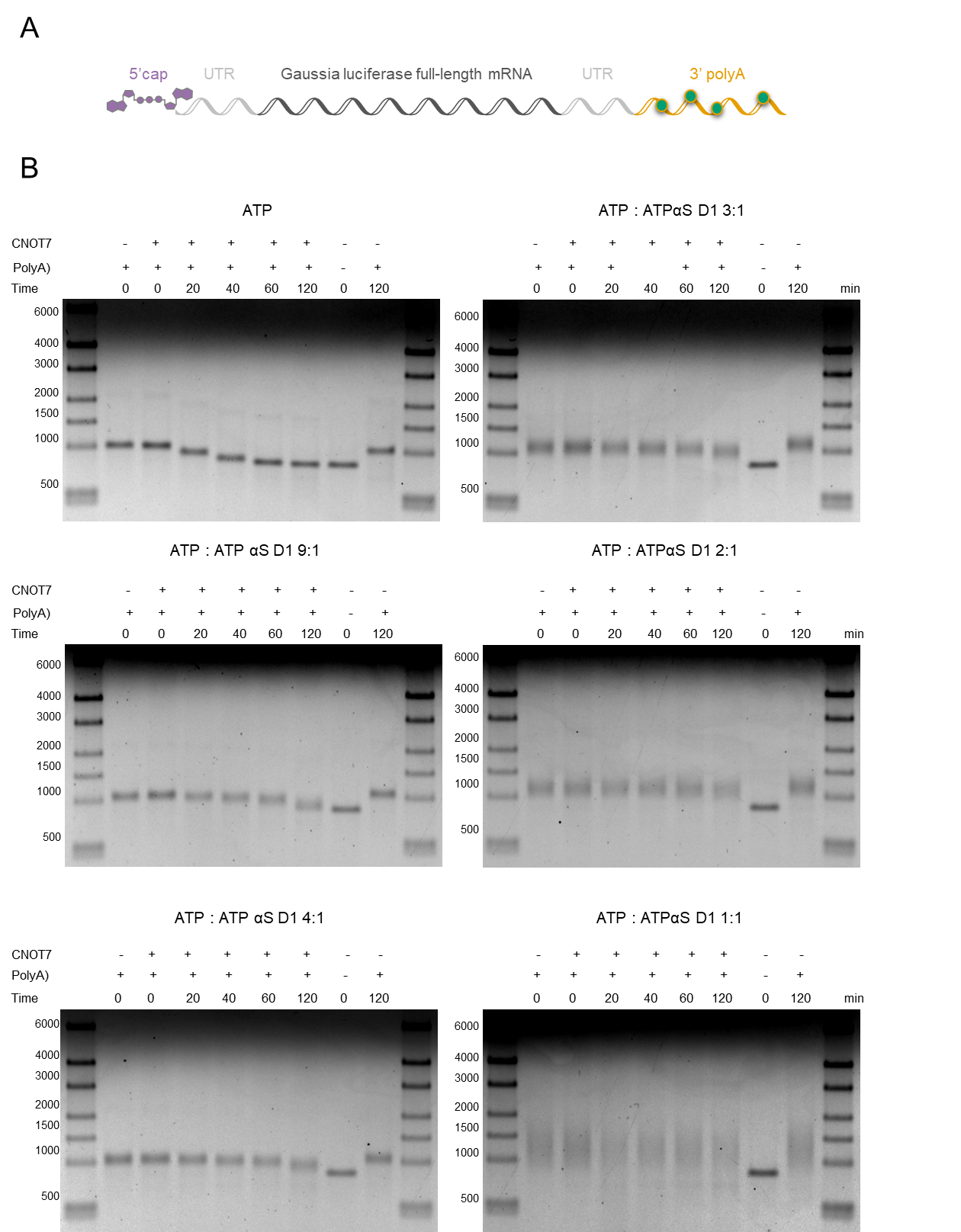


*Figure S12. Analysis of RNA G1 susceptibility to deadenylation. (A) Schematic representation of Gaussia luciferase mRNA (RNA G1) used for degradation assay with human CNOT7 deadenylase. (B) Third repetition of stability analysis with HPLC purified RNA G polyadenylated by PAP and ATP or the mix of ATP:ATPαS D1 at 9:1, 4:1, 3:1, 2:1 or 1:1 molar ratio. RNA G1 containing unmodified polyA tail (marked as ATP or 10:0) served as a positive control of deadenylation. All variants of RNA G1 were incubated with CNOT7 deadenylase for 0-120 min. The reaction was stopped by adding equal volume of loading dye containing 50 mM EDTA, and the polyA tail degradation rate was analyzed on 1% 1×TBE agarose gel stained with ethidium bromide. The lengths of digested polyA tails were estimated using Image Lab 6.0.1 Software (Bio-Rad). The polyA tails degradation rates were estimated as a number of nucleosides removed by CNOT7 deadenylase during the reaction period.*


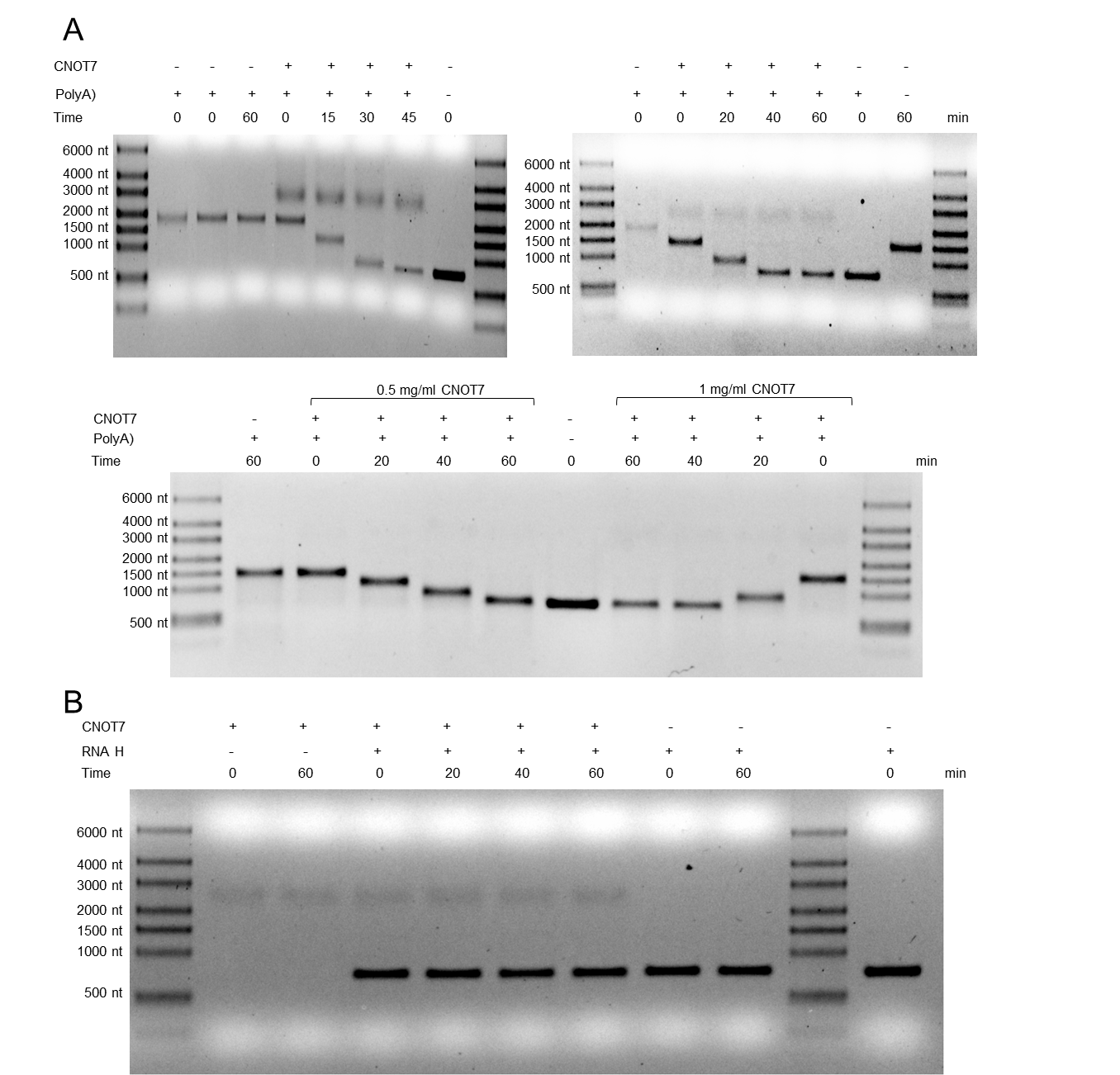


*Figure S13. Optimization of deadenylation reaction and verification of human CNOT7 deadenylase substrate specificity. (A) 50 ng (left panel), 80 ng (right panel) or 40 ng (bottom panel) of unmodified RNA H1 (RP HPLC-purified, polyadenylated with ATP by PAP, phenol:chloroform extrancted and isopropanol precipitated) was used as a substrate for CNOT7 deadenylase. RNA H1 was incubated at 37 °C in presence of 1 mg/mL of CNOT7 for 0, 15, 30 and 45 min (left panel), 0, 20, 40 and 60 min (right panel) or with 0.5-1 mg/mL CNOT7 for 0, 20, 40 and 60 min (bottom panel). Deadenylation reaction was stopped by adding equal volume of loading dye containing 50 mM EDTA. Half of the reaction volume was loaded on 1×TBE 1% agarose gel. (B) 80 ng of RNA H1 was incubated in presence of 1 mg/mL of CNOT7 deadenylase for 0, 20, 40 and 60 min at 37 °C. Enzyme was inhibited by adding equal volume of loading dye containing 50 mM EDTA. Half of the reaction volume was loaded on 1×TBE 1% agarose gel. As a control reactions samples with CNOT7 or RNA H alone were incubated for 0 min and 60 min.*


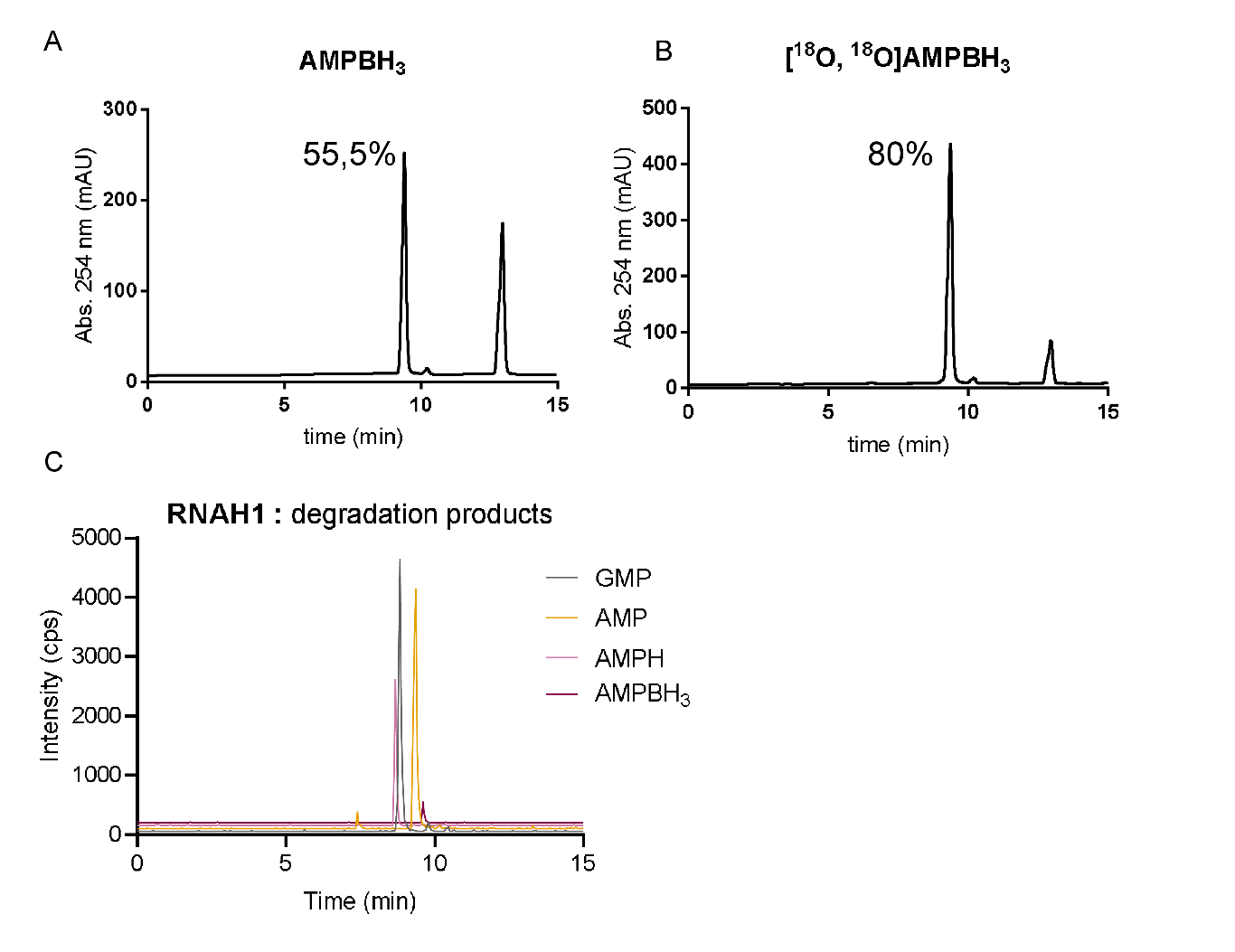


*Figure S14. Chemical and enzymatically instability of boranophosphate nucleotides and RNA (A) HPLC purity of AMPBH_3_ and isotopically labeled analogue (B) after 1 day in solution. (C) products obtained after enzymatic degradation of RNA H1 using mix of SVPDE and CNOT7*


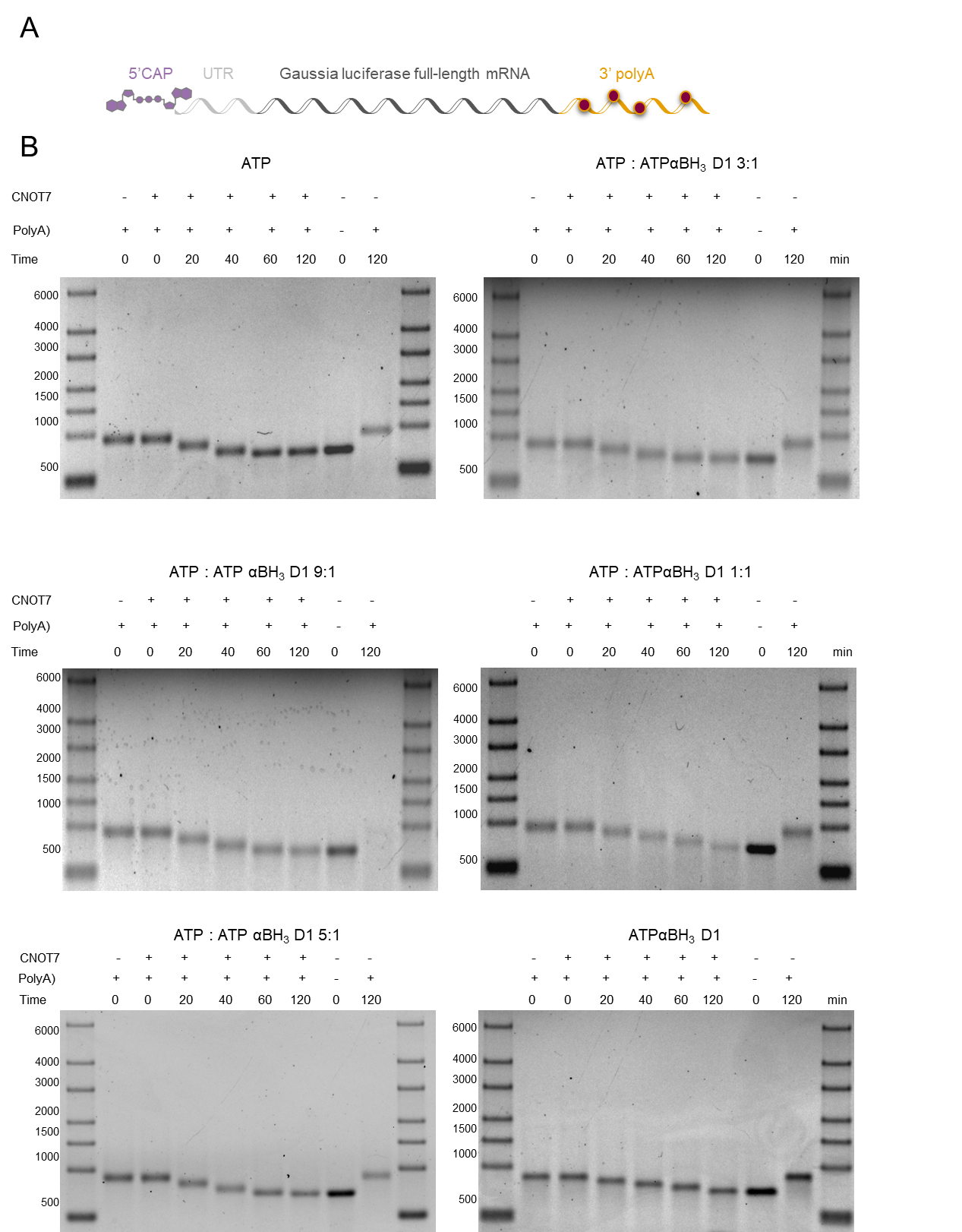


*Figure S15. Analysis of RNA H1 susceptibility to deadenylation. (A) Schematic representation of Gaussia luciferase mRNA (RNA H1) used for degradation assay with CNOT7 deadenylase. (B) First repetition of stability analysis with HPLC purified RNA H polyadenylated by PAP and ATP, ATPαBH_3_ D1 or the mix of ATP:ATPαBH_3_ D1 at 9:1, 5:1, 3:1 or 1:1 molar ratio. RNA H1 containing unmodified polyA tail (marked as ATP or 10:0) served as a positive control of deadenylation. All variants of RNA H1 were incubated with CNOT7 deadenylase for 0-120 min. The reaction was stopped by adding equal volume of loading dye containing 50 mM EDTA, and the polyA tail degradation rate was analyzed on 1% 1×TBE agarose gel stained with ethidium bromide. The lengths of digested polyA tails were estimated using Image Lab 6.0.1 Software (Bio-Rad). The polyA tails degradation rates were estimated as a number of nucleosides removed by CNOT7 deadenylase during the reaction period.*


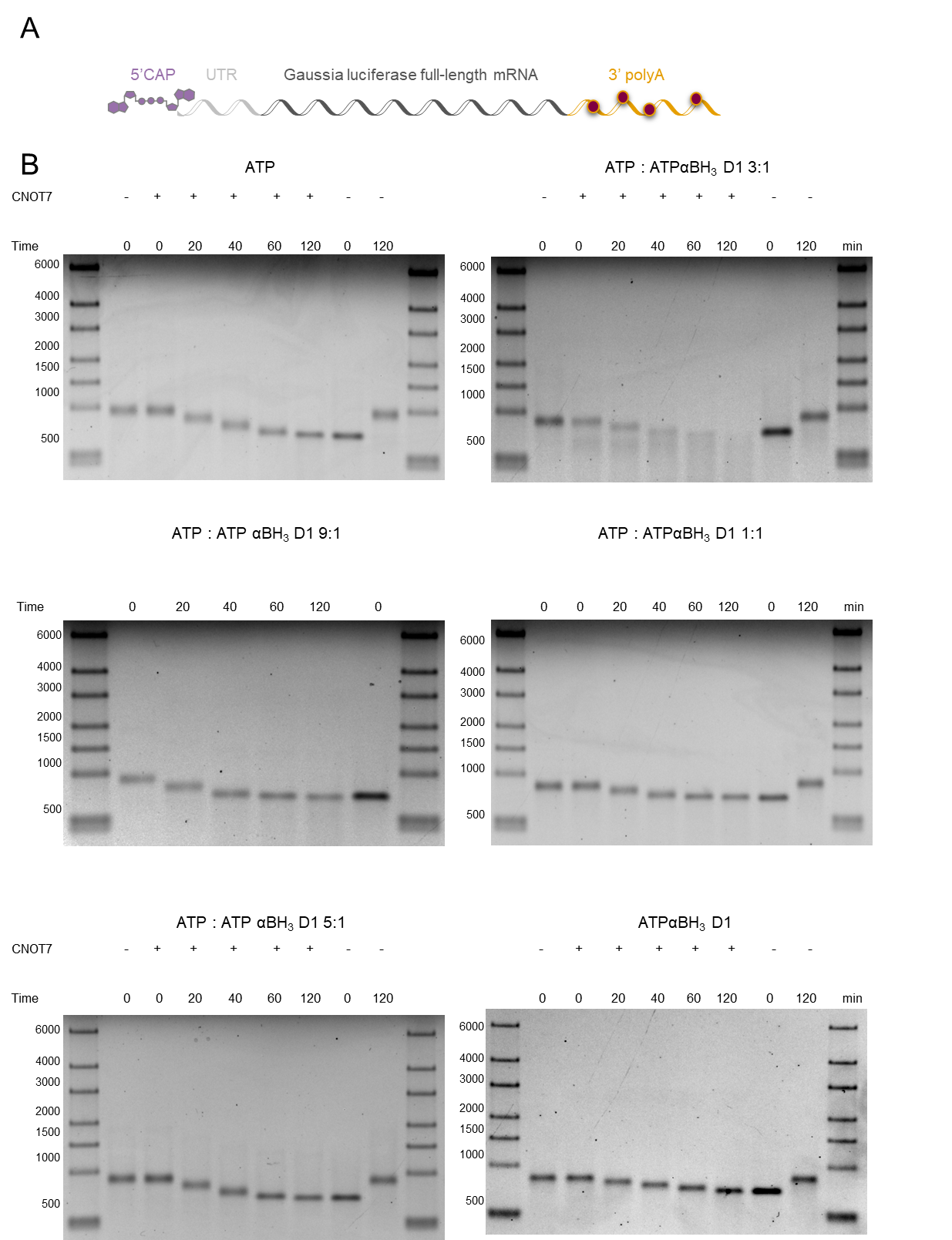


*Figure S16. Analysis of RNA H1 susceptibility to deadenylation. (A) Schematic representation of Gaussia luciferase mRNA (RNA H1) used for degradation assay with CNOT7 deadenylase. (B) Second repetition of stability analysis with HPLC purified RNA H polyadenylated by PAP and ATP, ATPαBH_3_ D1 or the mix of ATP:ATPαBH_3_ D1 at 9:1, 5:1, 3:1 or 1:1 molar ratio. RNA H1 containing unmodified polyA tail (marked as ATP or 10:0) served as a positive control of deadenylation. All variants of RNA H1 were incubated with CNOT7 deadenylase for 0-120 min. The reaction was stopped by adding equal volume of loading dye containing 50 mM EDTA, and the polyA tail degradation rate was analyzed on 1% 1×TBE agarose gel stained with ethidium bromide. The lengths of digested polyA tails were estimated using Image Lab 6.0.1 Software (Bio-Rad). The polyA tails degradation rates were estimated as a number of nucleosides removed by CNOT7 deadenylase during the reaction period.*


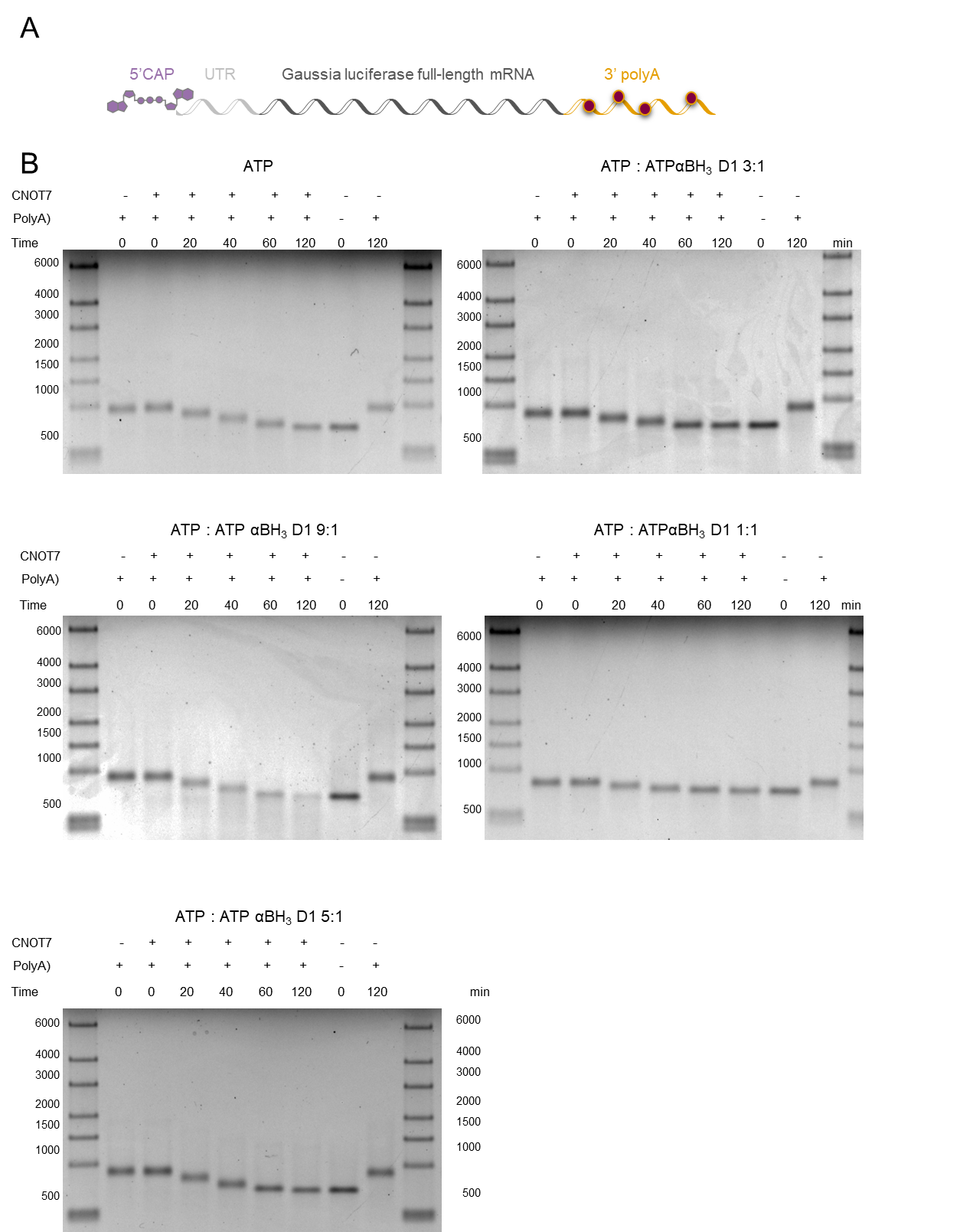


*Figure S17. Analysis of RNA H1 susceptibility to deadenylation. (A) Schematic representation of Gaussia luciferase mRNA (RNA H1) used for degradation assay with CNOT7 deadenylase. (B) Third repetition of stability analysis with HPLC purified RNA H polyadenylated by PAP and ATP, ATPαBH_3_ D1 or the mix of ATP:ATPαBH_3_ D1 at 9:1, 5:1, 3:1 or 1:1 molar ratio. RNA H1 containing unmodified polyA tail (marked as ATP or 10:0) served as a positive control of deadenylation. All variants of RNA H1 were incubated with CNOT7 deadenylase for 0-120 min. The reaction was stopped by adding equal volume of loading dye containing 50 mM EDTA, and the polyA tail degradation rate was analyzed on 1% 1×TBE agarose gel stained with ethidium bromide. The lengths of digested polyA tails were estimated using Image Lab 6.0.1 Software (Bio-Rad). The polyA tails degradation rates were estimated as a number of nucleosides removed by CNOT7 deadenylase during the reaction period.*

# Experimental procedures

## Synthesis of reference compounds and internal standards

**Nucleoside 5’-O-monophosphates: AMP, [^18^O,^18^O]AMP, GMP, [^18^O,^18^O]GMP**

Nucleoside 5′-phosphorylation was essentially performed as described earlier(1), (Figure S2A). Briefly, an appropriate nucleoside (0.01 mmol) was dissolved/suspended in trimethyl phosphate (0.5 mL) at 0 °C followed by the addition of phosphoryl chloride (POCl_3_, 0.03 mmol). The reaction was monitored by RP HPLC (as described above in General information). The reaction was quenched with either 97% [^18^O]-H_2_O or H_2_O, when the conversion of the substrate reached 60–90%. pH was neutralized using NaHCO_3_.The desired nucleoside 5′-monophosphate was purified by RP HPLC. The purity and isotopic composition of the isolated products were analyzed by electrospray ionization (ESI,−)/MS (Table S7). The analyses confirmed that the products obtained via hydrolysis with heavy water contained at least 87% of the expected [^18^O,^18^O] double-labeled nucleoside 5′-monophosphate along with smaller amounts (12%) of [^18^O] single-labeled product, and less than 1% of unlabeled product.

**Adenosine 5’-O-monothiophosphate: AMPS, [^18^O,^18^O]AMPS**

Nucleoside 5′-thiophosphorylation was essentially performed as described earlier (2), (Figure S2A). Briefly, adenosine (0.01 mmol) was suspended in trimethyl phosphate (0.5 mL) and placed in an ice-bath for 5 min, followed by addition of 2,6-lutidine (0.04 mmol) and thiophosphoryl chloride (PSCl_3_, 0.03 mmol). The reaction progress was monitored by RP HPLC. The reaction was quenched after 6 h with either 97% [^18^O]-H_2_O or H_2_O, when conversion of the substrate reached 60–90%. pH was neutralized using NaHCO_3_.The products were purified by RP HPLC (as described above in General information). The purity and isotopic composition of the isolated products was analyzed by ESI(−)/MS (Table S7). The analyses confirmed that the products obtained via hydrolysis with heavy water contained at least 87% of the expected [^18^O,^18^O] double-labeled nucleoside 5′-monophosphate along with smaller amounts (11%) of [^18^O] single-labeled product, and less than 2% of unlabeled product.

**Adenosine-5’-O-(*H-*phosphonate): AMPH, [^18^O,^18^O] AMPH**

AMPH and [^18^O,^18^O] AMPH were synthesized as previously described (3) (Figure S2B). 2´,3´-O-isopropylidene-adenosine (0.65 mmol) was suspended in trimethyl phosphate (3 mL) and placed in an ice-bath followed by the addition of phosphorus trichloride (PCl_3_, 1.95 mmol). Reaction progress was monitored by RP HPLC and quenched with 97% [^18^O]-H_2_O or H_2_O after 0.5 h. Then, sodium bicarbonate (NaHCO_3_) was added to neutralize the pH, and the solution was kept for 6 h at RT to remove the isopropylidene protecting group. The product was purified by HPLC (as described above in General information). The purity and isotopic composition of the isolated products was analyzed by ESI(−)/MS (Table S7). The analyses confirmed that the products obtained via hydrolysis with heavy water contained at least 84% of the expected [^18^O,^18^O] double-labeled nucleoside 5′-monophosphate along with smaller amounts (10%) of [^18^O] single-labeled product, and less than 6% of unlabeled product.

**Adenosine 5’-O-monoboranophosphate: AMPBH_3_, [^18^O,^18^O] AMPBH_3_**

AMPBH_3_ and [^18^O,^18^O] AMPBH_3_ were obtained as previously described (3). Adenosine-5’-O-(*H-*phosphonate) or [^18^O,^18^O] adenosine-5’-O-(*H-*phosphonate) (0.06 mmol) was placed in a rubber septum capped round-bottom flask under argon atmosphere and suspended in acetonitrile (1 mL). Then, N,O-bis(trimethylsilyl)acetamide (BSA, 1.2 mmol) was added through a syringe and after 30 min of stirring, BH3·SMe_2_ (0.12 mmol) was added and the mixture was left for 30 min. An adequate product was released after addition of a trimethylamine (TEA)/methanol solution. The solution was evaporated to dryness and three times re-evaporated from methanol. Products were purified by HPLC (as described above in General information). The purity and isotopic composition of the isolated products was analyzed by ESI(−)/MS (Table S7). The analyses confirmed that the products obtained via hydrolysis with heavy water contained at least 99% of the expected [^18^O,^18^O] double-labeled nucleoside 5′-monophosphate.

**8-Deuteroadenosine 5’-O-triphosphate: ^D^ATP**

Adenosine 5’-*O*-triphosphate sodium salt (3 mg) was diluted in 1 mL of D_2_O and TEA was added to pH 10. The mixture was incubated at 60 °C. The progress of gradual H-to-D exchange was monitored by HPLC-MS. After 2 d, the sample was freeze-dried, redissolved in D_2_O in the presence of TEA, and the incubation was continued at 60 °C. The procedure was repeated two more times (after 2 and 4 days). After 6 d total incubation time, the exchange efficiency reached 94%. The product was purified by HPLC and freeze-dried three times.

## Solid-phase synthesis of oligoribonucleotide 5’-phosphate pG(CU)_5_A_11_ used as a control sample (RNA C, Table 1)

Solid-phase synthesis of oligoribonucleotide was performed on a 1 μmol scale using AKTA Oligopilot plus 10 synthesizer (GE Healthcare) and Custom PrimerSupport™ Ribo A 40 (GE Healthcare) solid support. In the coupling step, 20 equivalents of N6-Benzoyl-2'-O-tert-butyldimethylsilyl-5'-O-DMT-adenosine 3'-CE (2’-*O*-TBDMS) phosphoramidate (rA^Ac^, rC^Ac^, rG^Ac^, U or biscyanoethyl phosphoramidite, all from ChemGenes) and 0.30 M 5-(benzylthio)-1-*H*-tetrazole in acetonitrile were recirculated through the column for 15 minutes. A solution of 3% (v/v) dichloroacetic acid in toluene was used as a detritilation reagent, 0.05 M iodine in pyridine/water (9:1) for oxidation, 20% (v/v) *N*-methylimidazole in acetonitrile as Cap A and a mixture of 40% (v/v) acetic anhydride and 40% (v/v) pyridine in acetonitrile as Cap B. After the last cycle of synthesis, RNA, still on the solid support, was treated with 20% (v/v) diethylamine in acetonitrile to remove the 2-cyanoethyl protecting groups. The product was cleaved from the solid support and deprotected with AMA (methylamine/ammonium hydroxide 1:1_v/v_; 55 °C, 1 h), evaporated to dryness and redissolved in dimethyl sulfoxide (100 μL). The TBDMS groups were removed using triethylammonium trihydrofluoride (TEA·3HF; 125 μL, 65 °C, 3 h) and the oligonucleotide was desalted by precipitation with sodium acetate and 1-butanol. Finally, the product was purified by RP HPLC (linear gradient of acetonitrile in ammonium acetate (CH_3_COONH_4_, pH 5.9) to give, after repeated freeze-drying from water, an ammonium salt of pG(CU)_5_A_11_. For LC-MS/MS analysis, 20 ng oligoRNA was digested using SVPDE, according to the above conditions.

## Purification of human CNOT7 deadenylase

The cDNA region encoding full length human CNOT7 deadenylase was subcloned into pET51b-derived expression vector with an N-terminal StrepTAGII-HA and C-terminal 10xHIS tag. Fusion protein was expressed in *E.coli* BL21(DE3) CodonPlus-RIL (Stratagene) in Super Broth Auto Induction Media (Formedium, Norfolk, UK) supplemented with ampicilin (50 µg/mL) for 48 h at 18 °C. Cells were spin down at 4000 × g at 4 °C for 15 min and stored in -20 °C. For purification, cells from 2 L of culture (60 g) were thawed, resuspended in 180 ml of lysis buffer (150 mM NaCl, 50 mM Tris pH 8.0, 10 mM imidazole, 10 mM β-mercaptoethanol, 300 mM urea, 50 mM arginine, 50 mM glutamic acid) supplemented with protease inhibitors (PMSF, pepstatin A, chymostatin, leupeptin, benzamidine) and lysosyme (0.25 mg/mL) and disrupted with EmulsiFlex-C3 high pressure homogenizer (Avestin Europe GmbH, Mannheim, Germany). Cell extract was clarified by ultracentrifugation at 140 000 x g at 4 °C for 45 min and recombinant CNOT7 protein was purified automatically using ÄKTAxpress FPLC system (GE Healthcare). The following protocol was used for automated purification: (1) nickel affinity chromatography (buffer: 150 mM NaCl, 10 mM Tris pH 8.0, 10 mM β-mercaptoethanol, 10 mM or 600 mM imidazole); (2) size-exclusion chromatography (buffer: 150 mM NaCl, 10 mM Tris pH 8.0; column: HiLoad 16/60 Superdex 200). Purity of CNOT7 deadenylase was validated on 12% PAGE. Purified protein was concentrated by ultrafiltration to 110-120 μM, flash-frozen with liquid nitrogen in storage buffer (SEC buffer supplemented with 15% glycerol and 1 mM DTT) and stored at -80 °C.

# References

1. Yoshikawa, M., Kato, T. and Takenishi, T. (1967) A novel method for phosphorylation of nucleosides to 5'-nucleotides. *Tetrahedron Letters*, 5065-5068.

2. Eckstein, F. (1970) Nucleoside phosphorothioates. *Journal of the American Chemical Society*, **92**, 4718-4723.

3. Kowalska, J., Wypijewska del Nogal, A., Darzynkiewicz, Z.M., Buck, J., Nicola, C., Kuhn, A.N., Lukaszewicz, M., Zuberek, J., Strenkowska, M., Ziemniak, M. *et al.* (2014) Synthesis, properties, and biological activity of boranophosphate analogs of the mRNA cap: versatile tools for manipulation of therapeutically relevant cap-dependent processes. *Nucleic Acids Research*, **42**, 10245-10264.
